# Chemoattraction and Recruitment of Activated Immune Cells, Central Autonomic Control, and Blood Pressure Regulation

**DOI:** 10.3389/fphys.2019.00984

**Published:** 2019-08-02

**Authors:** Khalid Elsaafien, Willian S. Korim, Anthony Setiadi, Clive N. May, Song T. Yao

**Affiliations:** ^1^Discovery Science, Florey Institute of Neuroscience and Mental Health, Parkville, VIC, Australia; ^2^Florey Department of Neuroscience and Mental Health, The University of Melbourne, Parkville, VIC, Australia

**Keywords:** neuroinflammation, chemoattraction, immune system, autonomic nervous system, hypertension

## Abstract

Inflammatory mediators play a critical role in the regulation of sympathetic outflow to cardiovascular organs in hypertension. Emerging evidence highlights the involvement of immune cells in the regulation of blood pressure. However, it is still unclear how these immune cells are activated and recruited to key autonomic brain regions to regulate sympathetic outflow to cardiovascular organs. Chemokines such as C-C motif chemokine ligand 2 (CCL2), and pro-inflammatory cytokines such as tumor necrosis factor alpha (TNF-α) and interleukin 1 beta (IL-1β), are upregulated both peripherally and centrally in hypertension. More specifically, they are upregulated in key autonomic brain regions that control sympathetic activity and blood pressure such as the paraventricular nucleus of the hypothalamus and the rostral ventrolateral medulla. Furthermore, this upregulation of inflammatory mediators is associated with the infiltration of immune cells to these brain areas. Thus, expression of pro-inflammatory chemokines and cytokines is a potential mechanism promoting invasion of immune cells into key autonomic brain regions. In pathophysiological conditions, this can result in abnormal activation of brain circuits that control sympathetic nerve activity to cardiovascular organs and ultimately in increases in blood pressure. In this review, we discuss emerging evidence that helps explain how immune cells are chemoattracted to autonomic nuclei and contribute to changes in sympathetic outflow and blood pressure.

## Introduction

The autonomic nervous system plays a major role in blood pressure regulation whereby dysfunction can lead to hypertension. Brain regions lacking a functional blood-brain barrier (BBB), known as circumventricular organs (CVOs), can sense and respond to circulating factors such as blood-borne hormones, like angiotensin II. This can lead to elevated sympathetic discharge and blood pressure (BP) (Nunes and Braga, 2011; Zubcevic et al., 2017). CVOs, such as the subfornical organ (SFO) and the area postrema (AP), regulate sympathetic outflow by changing the activity of neurons in the paraventricular nucleus of the hypothalamus (PVN) and the rostral ventrolateral medulla in the brainstem (RVLM) (van der Kooy and Koda, 1983; Dampney et al., 1987; Braga et al., 2011). Both the PVN and the RVLM are implicated in the regulation of sympathetic outflow to cardiovascular organs *via* direct projections to sympathetic preganglionic neurons (SPNs) located in the spinal cord (Strack et al., 1989a,b; Schramm et al., 1993). Thus, these brain regions play an important role in regulating homeostatic levels of sympathetic outflow to the cardiovascular organs such that activation of the AP, by inflammatory mediators such as tumor necrosis factor alpha (TNF-α), for example, can lead to increases in both cardiac and renal sympathetic nerve activity (Korim et al., 2018).

The upregulation of pro-inflammatory mediators is associated with human hypertension (Chrysohoou et al., 2004; Antonelli et al., 2012). Studies in experimental rodent models of hypertension confirm this finding. Moreover, they further show that upregulation of a wide range of pro-inflammatory mediators occurs in key brain regions known to regulate sympathetic outflow to cardiovascular organs (Shen et al., 2015). These pro-inflammatory cytokines include TNF-α, interleukin 1 beta (IL-1β), interleukin 6 (IL-6), and pro-inflammatory chemokines such as C-C motif chemokine ligand 2 (CCL2). These mediators are upregulated in the PVN and RVLM, in models of both primary and secondary hypertension (Li et al., 2014; Song et al., 2014). Selective blockade of these inflammatory mediators in the central nervous system reduces BP in animal models of hypertension (Li et al., 2014; Song et al., 2014). These studies indicate that upregulation of pro-inflammatory mediators in brain regions that control cardiovascular function contributes to sustained BP increase in hypertension. However, the factors leading to upregulation of these mediators in critical brain areas in the context of cardiovascular control remain unclear.

Recently, focus has shifted to the role of immune cells in the development of hypertension (Bomfim et al., 2018; Caillon et al., 2018; Carnagarin et al., 2018). Resident immune cells in the central nervous system (microglia) are responsible for local inflammatory processes in the brain (Shen et al., 2015). In fact, chronic central infusion of minocycline, an anti-inflammatory antibiotic that reduces microglia activation, reduces central inflammation and BP in hypertension (Shi et al., 2010). There is also evidence that peripheral bone marrow immune cells are involved in inducing brain inflammation, leading to a hypertensive phenotype (Santisteban et al., 2015). Interestingly, when the bone marrow of spontaneously hypertensive rats (SHRs) is ablated and replaced with bone marrow from normotensive Wistar Kyoto rats (WKYs), central inflammation is attenuated, leading to BP reduction (Santisteban et al., 2015). This evidence suggests that peripheral immune cells play an important role in central inflammation and the development of hypertension.

Infiltrating immune cells are activated and recruited by pro-inflammatory chemokines, such as CCL2 (Deshmane et al., 2009). Interestingly, CCL2 is upregulated in the PVN of hypertensive rodents. This upregulation of CCL2 was linked to the presence of infiltrating immune cells in the PVN of these animals (Wang et al., 2018). Moreover, there is a clear gradient of CCL2 levels in hypertensive animals, whereby the lowest levels are detected in bone marrow and the highest levels are detected in the cerebrospinal fluid (CSF) of rodents (Santisteban et al., 2015). Hence, this forms a distinct chemotactic gradient, such that immune cells are recruited to specialized cardiovascular control regions of the brain. Once there, they initiate an inflammatory cascade, which impairs sympathetic control and mediates sustained increases in BP.

In this review, we discuss the evidence supporting brain chemoattraction and the involvement of immune cells in regulating sympathetic outflow to cardiovascular organs. We will focus on the effects of chemoattraction of immune cells to induce inflammatory cascades in key autonomic brain centers that control cardiovascular function, and the potential role of these changes in the development of hypertension.

## Upregulation of Brain Pro-Inflammatory Cytokines and Chemoattraction of Immune Cells on the Regulation of Blood Pressure

### Upregulation of Brain Pro-inflammatory Cytokines in Hypertension

Increases in BP in rodent models of hypertension are associated with the upregulation of pro-inflammatory mediators, both peripherally and centrally. Recent studies demonstrate that a number of pro-inflammatory mediators are elevated in different rodent models of hypertension (Jia et al., 2014; Song et al., 2014; Yu et al., 2015; Li et al., 2016). For example, there are significantly higher levels of TNF-α, IL-1β, IL-6, and CCL2 in the PVN of SHRs, a model of primary hypertension, compared with normotensive WKYs. Similarly, in a model of secondary hypertension (renovascular; two kidney-one clip, 2 K-1C), the levels of TNF-α, IL-1β, IL-6, and CCL2 are elevated in the RVLM (Li et al., 2014). Furthermore, the levels of these pro-inflammatory mediators are also elevated in the PVN of angiotensin II-induced hypertensive rat models (Kang et al., 2009, 2014; Sriramula et al., 2013; Su et al., 2014), as well as in high salt diet-induced hypertension (Gao et al., 2016; Wang et al., 2018). The downstream effect of this increased inflammation is thought to contribute to altered neuronal signaling caused by imbalances in neurotransmitter and neuromodulator levels in key autonomic brain centers. For instance, glutamate and norepinephrine are upregulated whereas GABA was downregulated within both the PVN and the RVLM of hypertensive animals (Jia et al., 2014; Song et al., 2014; Yu et al., 2015; Li et al., 2016). Thus, changes in the activity of neurons in key autonomic brain nuclei may contribute to elevated sympathetic nerve activity (SNA) and increases in BP.

Blockade of receptors for pro-inflammatory mediators within cardiovascular brain regions reduces BP in rodent models of hypertension. Recently, we showed that blockade of TNF-α receptors (TNFR1) in the AP reduces BP in the 2 K-1C model of hypertension (Korim et al., 2018). Others have also demonstrated that non-selective blockade of TNF-α receptors (Sriramula et al., 2013; Song et al., 2014), IL-1β receptors (Lu et al., 2017), and the downstream secondary messenger of pro-inflammatory cytokines NF-κB (Yu et al., 2015) in the PVN of hypertensive rats reduced BP. Interestingly, antagonism of pro-inflammatory cytokine receptors within the central nervous system not only reduced SNA and BP in hypertension, but also appeared to restore the neurotransmitter imbalances and excessive activation of cardiovascular brain regions (Sriramula et al., 2013; Song et al., 2014; Lu et al., 2017). Thus, the dysregulation of pro-inflammatory mediator levels within key autonomic centers appears to be associated with the development of hypertension.

Exogenous application of pro-inflammatory cytokines into specific central cardiovascular control centers of normotensive animals increases SNA and BP. For example, microinjections of TNF-α and IL-1β into the SFO (Wei et al., 2015) and in the PVN (Shi et al., 2011) increased renal SNA and BP. Furthermore, our group has recently shown that the microinjection of TNF-α into the AP increases both renal and cardiac SNA and BP (Korim et al., 2018). Interestingly, we found receptors for TNF-α to be expressed on AP neurons projecting to the RVLM – a cardiovascular brain region known for containing neurons directly projecting to SPNs (Strack et al., 1989a,b; Schramm et al., 1993). These neurons appear to be chronically activated in hypertensive animals and are also active following microinjection of TNF-α into the AP (Korim et al., 2018). These studies provide a direct causal relationship whereby activation of neurons by pro-inflammatory mediators, within important central cardiovascular control regions such as the AP, increases the sympathetic outflow and BP. Indeed, we have also previously demonstrated that the AP is critical in driving the increased cardiac SNA in an ovine model of heart failure (Abukar et al., 2018). Taken together, these studies provide compelling evidence to support a link between increases in brain pro-inflammatory mediators and the dysregulation of SNA and BP in hypertension.

### C-C Motif Chemokine Ligand 2 and the Chemoattraction of Immune Cells in Hypertension

Chemokines are pro-inflammatory mediators and chemotactic cytokines, whose main function is to regulate cell trafficking (Rollins, 1997; Deshmane et al., 2009; Zlotnik and Yoshie, 2012). These proteins create a concentration gradient and activate immune cells, causing them to move up this chemotactic gradient (Zlotnik and Yoshie, 2012). The chemokine CCL2 (also known as monocyte chemoattractant protein-1 or MCP-1), and its cognate receptor C-C Chemokine receptor type 2 (CCR2), is one of the most extensively studied chemokines. While CCL2 can be secreted by a variety of cell types, including endothelial cells and vascular smooth muscle cells (Bartoli et al., 2001), the main source of CCL2 is believed to be monocytes/macrophages (Yoshimura et al., 1989a,b). CCL2 is secreted in response to injury, oxidative stress, growth factors, and expression of other pro-inflammatory cytokines – where CCL2 secretion forms a gradient toward these stimuli. This process is termed chemotaxis, where CCL2 recruits circulating monocytes/macrophages to the respective chemical stimulus in the inflamed tissue or site of injury (Ajuebor et al., 1998). Evidence that CCL2 plays a vital role in the process of monocyte recruitment and cytokine expression is demonstrated by the finding that these are abnormal in CCL2 knockout mice (Lu et al., 1998). Interestingly, both CCL2 and its receptor, CCR2, are expressed and produced in the brain, specifically in central autonomic control centers such as the PVN and the RVLM (Wittendorp et al., 2004; Banisadr et al., 2005; Hinojosa et al., 2011; Morioka et al., 2013). However, the extent to which CCL2 and the chemoattraction of immune cells contribute to increased SNA and BP in the development of hypertension is still unknown and requires further investigation.

There is some evidence suggesting the involvement of increased chemoattraction of immune cells by CCL2 into cardiovascular brain centers during the development of hypertension. For example, selective antagonism of CCR2 receptors reduces BP in rodent models of hypertension (Aiyar et al., 1999; Elmarakby et al., 2007; Chan et al., 2012; Chang et al., 2014; Wang et al., 2015). Furthermore, studies in models of both primary and secondary hypertension reveal upregulation of CCL2 both peripherally and centrally (Sriramula et al., 2013; Li et al., 2014; Song et al., 2014), with a 3-fold elevation in the levels of CCL2 within the PVN of hypertensive animals (Sriramula et al., 2013; Li et al., 2014; Song et al., 2014). Upregulation of CCL2 occurs in the bone marrow, serum, and cerebrospinal fluid of SHRs compared with normotensive WKYs (Santisteban et al., 2015). Interestingly, the increase in CCL2 levels in SHRs forms a gradient from the bone marrow (lowest concentrations) toward the cerebrospinal fluid (highest concentrations) (Santisteban et al., 2015), possibly forming a chemotactic gradient toward the central nervous system. Thus, it seems that CCL2 chemoattracts immune cells and triggers an inflammatory cascade within the brain, leading to increases in BP.

## The Neuro-Immune-Inflammatory Model of Hypertension

In hypertension, increased circulating levels of angiotensin II is a potential cause for increased levels of brain CCL2 (Matsuda et al., 2015). A hallmark of hypertension is the upregulation of renin-angiotensin system and increased levels of angiotensin II (Goldblatt et al., 1934; Crowley et al., 2006, 2010). While not all human essential hypertension is angiotensin II mediated, the serum levels of CCL2 are increased in hypertensive patients (Antonelli et al., 2012). Interestingly, treating these patients with angiotensin II receptor blockers, which reduces blood pressure, also reduces plasma levels of CCL2 (Marketou et al., 2011), suggesting a link between angiotensin II and increased levels of CCL2. Similarly, in a rodent renovascular model of hypertension, peripheral blockade of receptors for angiotensin II attenuates peripheral CCL2 production (Xie et al., 2006). Moreover, *in vitro* studies show that angiotensin II can directly stimulate the production of CCL2 from monocytes and vascular smooth muscle cells (Chen et al., 1998; Tsou et al., 2007). In addition, systemic angiotensin II infusion increases CCR2 receptor expression in circulating monocytes – which is blunted by blocking angiotensin II receptors (Ishibashi et al., 2004). Recent studies further show that application of angiotensin II to primary hypothalamic neurons induces increased CCL2 mRNA and CCL2 protein levels in the cell culture media (Santisteban et al., 2015). As such, the stimulation of receptors for angiotensin II on peripheral circulating monocytes, vascular smooth muscle cells, and even on neurons induces the production of CCL2 in these cells.

**Figure 1:**
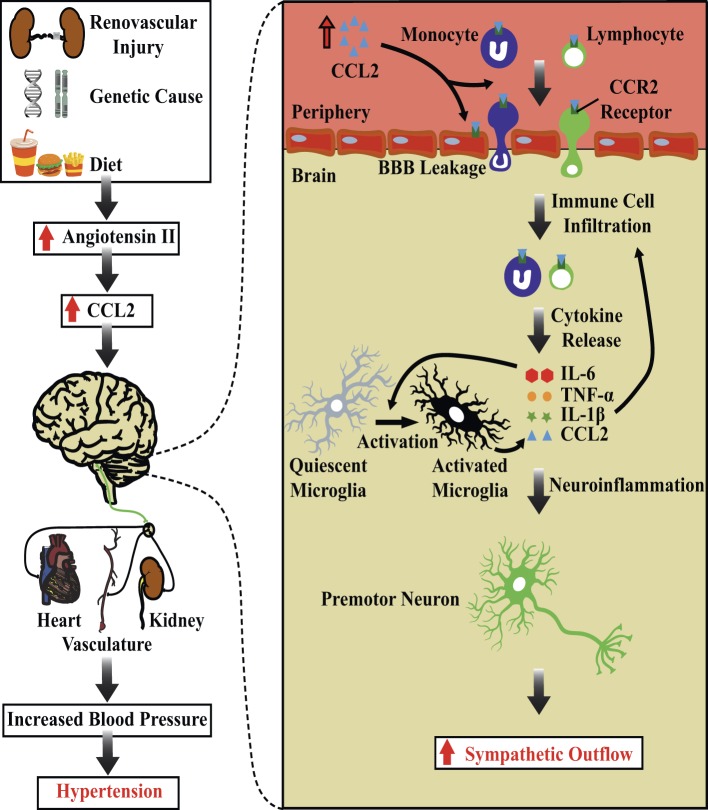
Chemoattraction of immune cells in the brain, autonomic dysfunction, and hypertension. Schematic diagram showing the potential role of chemoattraction of immune cells and their components in determining increases in sympathetic nerve activity and blood pressure. Increased circulating levels of angiotensin II leads to production of the chemokine CCL2. CCL2 can act directly on the BBB, increasing the permeability of endothelial cells and recruiting immune cells to the brain tissue. The resulting overexpression of pro-inflammatory cytokines produces sustained activation of cardiovascular sympathetic neurons and increases blood pressure. Abbreviations: BBB, blood brain barrier; CCL2, C-C motif chemokine ligand 2; CCR2, C-C Chemokine receptor type 2; IL-1β, interleukin 1 beta; IL-6, interleukin 6; TNF-α, tumor necrosis factor alpha.

Increased levels of CCL2 lead to the disruption of the BBB and facilitate immune cell infiltration into the brain tissue. While the BBB prevents immune cells from entering the brain, we have previously suggested that this structure is disrupted in hypertension (Setiadi et al., 2018). The regulation of BBB permeability involves tight junction proteins expressed on endothelial cells (Begley and Brightman, 2003). CCL2 is known to disrupt the BBB, through dysregulation of tight junction proteins such as ZO-1, ZO-2, occludin, and claudin-5 (Stamatovic et al., 2009; Roberts et al., 2012). *In vitro* studies have demonstrated that the application of CCL2 to primary mouse brain endothelial cell cultures reorganizes and redistributes tight junction proteins, increasing the permeability of the BBB (Stamatovic et al., 2003, 2009). Hence, CCL2 can directly disrupt the BBB by regulating the distribution of tight junction proteins. In SHRs, increased BBB permeability in the PVN and RVLM facilitates the entry of circulating angiotensin II into these brain structures (Biancardi et al., 2013). Interestingly, *in vitro* studies using primary human brain endothelial cells corroborate *in vitro* animal studies by showing that CCL2 can disrupt tight junction proteins expressed on endothelial cells cultured from human brains (Roberts et al., 2012). Furthermore, the application of CCL2 to primary human brain endothelial cell cultures induces the expression of cell adhesion molecules, such as PECAM-1 on the surface membrane of endothelial cells, which is required for facilitating transmigration of immune cells through endothelial cells (Muller et al., 1993; Roberts et al., 2012). Thus, not only can CCL2 reorganize the distribution of tight junction proteins expressed on endothelial cells to increase BBB permeability, it can also induce the expression of cell adhesion molecules on the surface membrane of endothelial cells to facilitate immune cell entry into brain tissue (Figure 1).

The recruitment and infiltration of immune cells into distinct brain regions can induce an inflammatory cascade resulting in the local upregulation of pro-inflammatory cytokines. The recruitment of activated immune cells into brain areas, including the PVN, results in the production and the upregulation of pro-inflammatory cytokines such as TNF-α, IL-1β, and IL-6 (Santisteban et al., 2015; Zubcevic et al., 2017; Sharma et al., 2019). These pro-inflammatory cytokines are capable of directly activating neurons and increasing sympathetic outflow and BP (Shi et al., 2011; Wei et al., 2015; Korim et al., 2018). Furthermore, the upregulation of pro-inflammatory cytokines and chemoattraction of activated immune cells also leads to activation of microglial cells (Santisteban et al., 2015; Zubcevic et al., 2017; Sharma et al., 2019). As microglia are the resident immune cells of the brain, activation of microglia leads to further release of pro-inflammatory mediators (Shi et al., 2010; Shen et al., 2015), therefore establishing an inflammatory state and escalating the inflammatory process centrally. Such a chronic inflammatory state results in further activation of immune cells, further neuroinflammation, and further rises in BP, leading to severe hypertension (Marvar et al., 2010). Whereas the blockade of receptors for pro-inflammatory cytokines in the brain (Sriramula et al., 2013; Song et al., 2014; Lu et al., 2017) prevents the activation of microglia (Shi et al., 2010), the recruitment of peripheral macrophages to the brain (Santisteban et al., 2015) and completely reverses the increased levels of peripheral and central pro-inflammatory cytokines and chemokines, leading to a reduced BP in hypertensive rats.

In summary, we propose a possible mechanism by which an inflammatory state in brain areas that control cardiovascular function is established, resulting in impaired BP control and hypertension. We propose that the increased levels of angiotensin II in the circulation results in the production and release of CCL2. This chemokine produces a chemotactic gradient that recruits immune cells toward the central nervous system. In addition, CCL2 increases BBB permeability and promotes the recruitment of activated immune cells. These immune cells initiate an inflammatory cascade where several pro-inflammatory mediators are released locally. Pro-inflammatory mediators activate neurons directly or indirectly by involving microglial transmission, which relays excitatory synapses to cardiovascular sympathetic premotor neurons in the ventrolateral medulla (Brown and Guyenet, 1985). The excitation of these neuronal subsets increases SNA and BP. These findings suggest that sustained activation of autonomic circuits contributes to the development of increased SNA and BP in neurogenic hypertension. Hence, chronic chemoattraction and recruitment of immune cells into key cardiovascular control regions might be a potential pathophysiological mechanism responsible for impaired BP control and hypertension (Figure 1).

## Conclusion

In this review, we discussed recent findings that support our proposal of a potential mechanism to explain the contribution of neuroinflammation and chemoattraction mediated by CCL2, in brain regions that control cardiovascular function, as a cause of the sustained increase in sympathetic tone and BP in hypertension. This mechanism likely involves the recruitment and the infiltration of immune cells by chemokines to key autonomic brain areas. In future, targeting brain immune cells or the chemoattraction of immune cells may serve as a new avenue for developing antihypertensive treatments. In fact, in the pre-clinical setting, blockade of receptors for CCL2 or preventing immune cells from being activated reduces BP, in addition to slowing the development of atherosclerosis and vascular hypertrophy (Aiyar et al., 1999; Bush et al., 2000; Elmarakby et al., 2007; Chan et al., 2012; Chang et al., 2014; Santisteban et al., 2015; Wang et al., 2015). However, more thorough investigations are required to determine the mechanism by which chemoattraction and immune cells interact with the central nervous system, during the development and maintenance of hypertension.

## Author Contributions

KE drafted the manuscript and Figure. WK, AS, CM, and SY critically revised the intellectual content. All authors conceived and discussed the content of the manuscript, approved the final version of the manuscript, and agreed to be accountable for all aspects of the work.

### Conflict of Interest Statement

The authors declare that the research was conducted in the absence of any commercial or financial relationships that could be construed as a potential conflict of interest.
